# Sexual Wellness: A Movement Happening Worldwide

**DOI:** 10.1055/s-0043-1777700

**Published:** 2023-12-23

**Authors:** Lucia Alves da Silva Lara

**Affiliations:** 1 Ribeirão Preto Medical School, São Paulo University, Ribeirão Preto, SP, Brazil

Sexual Health (SH) is an area that requires special attention, as it is directly related to sexual behavior, which has the potential to impact sexual and reproductive health. In 1994, in Cairo, the Program of Action of the International Conference on Population and Development included SH in the definition of reproductive health, 1 indicating that SH extends beyond reproductive care and counseling for sexually transmitted diseases, but aims to promote sexual pleasure, which is fundamental to human health. Thus, Sexual and Reproductive Health (SRH) became the area responsible for promoting SH from the perspective of pleasure, which is essential for physical and mental health, as well as for people's quality of life. A pleasurable sexual life promotes assertiveness in social and marital relationships, contributing to the longevity of interpersonal relationships. 2 Conversely, sexual dysfunctions negatively interfere with people's quality of life and mental health, being associated with conditions such as anxiety, emotional stress, depression, cardiovascular disease, chronic pelvic pain, among other. 3

Historically, issues related to sexual experience have been permeated by taboo, and in the clinical context, most Sexual and Reproductive Health (SRH) programs focus on contraception, teenage pregnancy, and sexually transmitted infections, which represent the tip of an iceberg supported by a broad base of biological, psychological, and environmental factors associated with risky sexual behaviors that lead to these pathologies. Each individual has their own motivations for seeking sexual pleasure; however, physiological sexual drive is a common motivation for most individuals. During puberty, the increase in androgens promotes an exacerbation of spontaneous sexual thoughts and an increase in interest in emotional relationships and sexual activity. 4 Sexual drive is the result of a complex motivation and reward mechanism elaborated in the limbic system (LS) and certain areas of the prefrontal córtex. 5 This mechanism is linked to certain neurotransmitters that act in the LS, such as the nucleus accumbens and the anteromedial preoptic area, which are associated with dopamine, playing a central role in the experience of reward and pleasure. These areas are connected to certain cortical regions of the central nervous system, which play a role in either positively or negatively modulating sexual impulse. 6

The motivation and reward mechanism plays a crucial role in habit formation and the repetition of motivating behaviors. Through this mechanism, the positive reinforcement of a pleasurable or rewarding experience, such as a pleasurable sexual activity, activates the memory of the pleasure sensation in specific areas of the LS, leading to the release of dopamine, creating a sense of reward and pleasure. This, in turn, activates intrinsic motivation to repeat the behavior that led to the reward. This mechanism is an essential part of what drives humans to seek pleasurable experiences and form habits, as it represents the connection between stimulus, behavior, and reward. 5 In experimental studies with animals, sex steroids testosterone, estrogen, and progesterone have a modulating effect on this sexual motivation mechanism. 6 Understanding the mechanism of motivation for seeking pleasure facilitates the comprehension that sexual pleasure is a natural and important aspect of the human experience.

Building upon older studies that have shown the benefits of sexual pleasure for human health, since 2008, the World Association for Sexual Health (WAS) has been urging both public and private academic institutions and society as a whole to recognize the connection between sexual pleasure, rights, and health. It emphasizes that sexual pleasure is a component of overall health and human well-being. 7 This has reinforced the importance of considering the experience of sexual pleasure in research, healthcare service delivery, and public policies. In Brazil, this movement was led by the Brazilian Federation of Gynecology and Obstetrics (FEBRASGO), which, through its Sexology Commission, developed a competency framework in sexual health for the training of Obstetrics and Gynecology resident physicians in the care of women with sexual complaints. 8 This document was validated by the Ministry of Education and Culture (MEC) in accordance with the Ministry of Health.

More recently, the global Sexual Wellness movement has been gaining momentum in the direction of promoting a holistic and positive approach to sexuality and SH. This movement embraces the idea that sexuality is a fundamental part of human life and should be addressed comprehensively, taking into account physical, emotional, psychological, and social aspects related to the development and experience of sexuality. Key points of this movement include sexual education, which is considered crucial to its agenda, open and healthy communication about sexuality in society at large, as well as the recognition and respect for sexual and gender diversity. It also advocates raising awareness about issues related to sexual health, consent, disease prevention, contraception, and more. 9

The discussion on the importance of sexual pleasure for the physical and mental health of individuals needs to be included on the public health agenda. Recognizing sexual pleasure as a vital component of human well-being is essential to address sexual health issues in a comprehensive and holistic manner. The Sexual Wellness movement calls for broader access to quality sexual health services for the population, ensuring that everyone has the opportunity to take care of their sexual health.
